# The Impact of Consumer Competence in Purchasing Foods on Satisfaction with Food-Related Consumer Policies and Satisfaction with Food-Related Life through Perceptions of Food Safety

**DOI:** 10.3390/foods9081103

**Published:** 2020-08-12

**Authors:** Hyun-Joo Lee

**Affiliations:** Department of Consumer Science, Inha University, Incheon 22212, Korea; hyunjoo.lee@inha.ac.kr; Tel.: +82-32-860-8118

**Keywords:** consumer competence, food safety, consumer policy, satisfaction with food-related life, knowledge-deficit model

## Abstract

Based on the knowledge-deficit model, this study proposes a relationship between consumer competence in purchasing foods and perceptions of the safety of imported and domestic foods. This study also examines how perceptions of the safety of imported and domestic foods affect satisfaction with food-related consumer policy and satisfaction with food-related life. Using data from the “2019 Consumer Behavior Survey for Food,” which has been conducted every year since 2013 by the Korea Rural Economic Institute, we analyzed the responses of a final sample of 5869 respondents. The hypothesized conceptual model was assessed through structural equation modeling. All but one of the proposed relationships between consumer competence in purchasing foods and perceptions of food safety were supported. The relationship between perceptions of food safety and satisfaction with food-related consumer policies depends on whether foods are imported or domestic. Food origin also affected the relationship between perceptions of food safety and satisfaction with food-related life. Satisfaction with food-related consumer policies is significantly connected with satisfaction with food-related life. We discuss how the findings of this study can be applied to the development of food-related consumer policies.

## 1. Introduction

Given that food is an important part of everyone’s daily life, the question “Is the food I consume safe?” is relevant to everyone. Furthermore, many people today find it more difficult to prepare and cook meals at home due to their busy daily lives, leading more people to turn to eating out, delivery food, or home-style foods [1,2]. Therefore, it has become more difficult for people to choose safe foods on their own [3]. As fewer people eat foods prepared at home, they are less certain that ingredients used in their food are safe. In this study, “safe” foods are defined as foods that do not contain biological, chemical, or physical substances that may harm consumers or threaten their health [4]. In addition, increased imports of foods from foreign countries has led to increased food safety issues [5]. Thus, it is necessary that the food industry prioritizes food safety and that consumer protection policy promotes it, as well as that consumers possess competence in purchasing safe foods.

Recent food safety incidents in Korea include hamburger disease, pesticide-tainted eggs, and salmonella-infected chocolate cake [6,7,8]. Consumers’ perceptions of food safety have fluctuated greatly because new food safety incidents have occurred each time the previous incident faded from consumers’ minds. Further, even though the government has renewed food-related consumer policy every year, no significant improvements have been made in food safety.

According to a survey of consumers’ perceptions of food safety conducted by a Korean government institution in the latter half of 2017, their overall sentiment index of food safety was 75.1%, a decrease of 9.5% from 84.6% in 2016 [9,10]. The index of food safety sentiment was determined by adding the percentages of respondents who answered that they perceived food safety management to be “neutral,” “safe,” or “extremely safe” [10]. The survey was conducted with both general panel members and expert panel members, and it is interesting that the two groups had different sentiments about food safety. Among the expert panel members, 48.3% felt that food was “safe” or “extremely safe,” but only 33.4% of the general panel members said it was “safe” or “extremely safe.” Meanwhile, 13% of the expert panel members answered “anxious” or “extremely anxious,” but 24.9% of the general panel members answered “anxious” or “extremely anxious” [10].

Consumers’ negative perceptions of food safety may be due to food safety incidents that have occurred frequently in recent years, but it may be also attributed to lack of consumer expertise or knowledge about food safety [11]. In other words, consumers may not have the expertise necessary to evaluate food safety correctly, so they may develop irrational attitudes toward food safety or show unreasonable responses, such as excessive psychological anxiety [11,12,13]. The knowledge-deficit model is a useful theory to explain the differences between experts and the general public in recognizing, evaluating, and coping with risk. The knowledge-deficit model, also known as the “knowledge gap” or “cognitive deficiencies” model, was originally used to describe public skepticism or hostility toward science, attributed to a lack of understanding derived from a lack of knowledge [14,15]. For example, Kellstedt et al. [16] used the knowledge-deficit model as a theoretical basis in their study of people’s perceptions of whether genetically modified foods are safe and found big differences between experts’ and non-experts’ perceptions. According to the knowledge-deficit model, increasing a person’s understanding and knowledge level can lead to a change in his or her attitudes and behaviors [15,17,18].

Based on the knowledge-deficit model, this study proposes a relationship between consumer competence in purchasing foods and perceptions of safety of imported and domestic foods. Although some researchers have examined the relationship between consumer competence and food safety, studies have shown inconsistent results across types of foods [19,20]. Thus, further investigation is needed. This study also examines how perceptions of safety of imported and domestic foods affect satisfaction with food-related consumer policy and satisfaction with food-related life. Knowledge is lacking about the relationships among food safety, consumer policy, and food-related life. Few studies have investigated how perceptions of food safety are related to other constructs such as satisfaction with consumer policy and food-related life. Whenever food safety accidents occur, the Korean government has attempted to secure public confidence and trust by establishing new food safety policy. For example, the Ministry of Food and Drug Safety (MFDS) has published a white paper every year since 2002 in which it reports the government’s policy achievements over the past year and suggests implementation plans for the coming year to apply and manage basic principles of food and drug safety and strengthen a response system to food and drug safety incidents [21]. The government has reacted sensitively and quickly to problems with food safety because such incidents can have a huge impact on society. Therefore, consumers’ perceptions of food safety can be used to predict their satisfaction with food-related consumer policy. This study also proposes that perceptions of food safety are a factor that determines satisfaction with food-related life. Therefore, this study will generate new insights into how food safety is important because food safety can help improve the quality of food-related consumer policy and life.

## 2. Theoretical Background

Agri-food consumption competence, a term based on the concept of consumer competence, refers to consumer competence in the context of agri-food consumption [22]. Agri-food consumption competence is needed for consumers in a rapidly changing market to have a reasonable selection of agri-foods, eat a safe and healthy diet, and fulfill their responsibilities [22,23]. Agri-food consumption competence is composed of three dimensions: “agri-food purchase competence,” “eating habit competence,” and “agri-food damage relief competence.” Agri-food purchase competence is a competence needed in purchasing agri-foods or food ingredients and choosing food options in restaurants. Eating habit competence is a competence needed for the cooking and ingestion of agri-foods. Agri-food damage relief competence is a competence related to the rights and responsibilities that consumers demand or should keep as citizens [23]. Each dimension of agri-food consumption competence can be divided further: agri-food purchase competence into “agri-food labeling,” “agri-food information,” and “agri-food purchase environment” competence; eating habit competence into “healthy eating,” “safe eating,” and “traditional eating” competence; and agri-food damage relief competence into “consumer rights and interests,” “consumer responsibility,” and “consumer problem solving” competence [22].

Food safety is defined as a condition in which risk or incidents relating to daily food consumption either cannot occur or cannot be ignored, regardless of the amount of food consumed, frequency of eating, and duration of eating [19,24]. van Rijswijk and Frewer [25] examined how consumers define food quality and food safety and found that they defined food safety in terms of “absence of risk,” “harmfulness,” or “health.” However, a number of participants could not provide an elaborate definition of food safety, possibly because food safety is a credence attribute (an attribute that consumers cannot be certain a product has, even after using or consuming the product) [26]. In the previous literature, perceptions of food safety have been discussed mainly in the context of perceived food safety, anxiety about food insecurity, and anxiety about food hazard, terms which have been interchangeably used [19,20,24].

The Korean government establishes consumer policy to address and solve the problems with which consumers are confronted. Consumer policy ultimately promotes consumers’ rights and interests and improves their living standards [27]. Food-related consumer policies are implemented primarily for the purpose of protecting consumers’ rights and improving consumer well-being [27]. Food-related consumer policies are intended to establish fair transaction order, ensure food safety, and allow consumers to practice a healthy food-related life. Food-related consumer policies can include policies relating to consumer education, public relations, information, and compensation for consumers affected by unsafe food. Thus, food-related consumer policies can be categorized into sub-policies involving transaction, safety, labeling, nutrition and diet, education and public relations, and compensation for affected consumers [27].

Satisfaction with food-related life is a concept originating from the concept of life satisfaction. It is defined as an overall assessment of a person’s food-related life (one of the life domains) involving “procurement, preparation, and consumption of food and meals according to his/her chosen criteria” [28]. Food-related life is one specific domain within a person’s whole life. Because food or diet is an essential for the quality of life, satisfaction with food-related life is an indispensable part of life satisfaction [29].

## 3. Conceptual Model and Hypotheses

**Figure 1:**
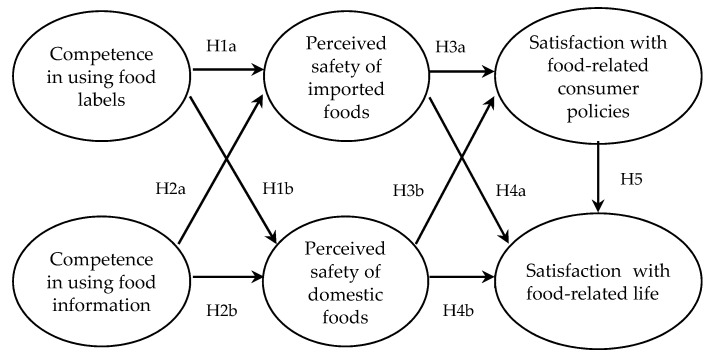
Conceptual model.

This study focuses on consumer competence in purchasing foods. In the previous study, in which both general consumers and experts were surveyed, consumer competence in purchasing foods was found to be the most important of the three dimensions of agri-food consumption competence [22]. In our proposed model, consumer competence in purchasing foods is conceptualized as a factor that influences perceptions of food safety, including both imported and domestic foods. In this study, food safety refers to a condition in which food will not harm consumers, because the risk of harm, such as biological, chemical, or physical substances in food that may threaten consumers’ health, has been reduced as much as possible [4]. The model also proposes that perceptions of food safety directly influence satisfaction with food-related life and indirectly influence satisfaction with food-related life through satisfaction with food-related consumer policies. Based on the reviews of previous literature, we propose the following relationships (see Figure 1).

### 3.1. The Relationship between Consumer Competence in Purchasing Foods and Perceptions of Food Safety

The knowledge-deficit model can be utilized as a theoretical basis for the relationship between consumer competence in purchasing foods and perceptions of food safety [13]. The theory assumes that a deficiency or discrepancy exists between scientists and lay people in terms of knowledge, awareness, access to information, and interest [13]. Filling this deficiency through education programs and information provision can lead to a change in attitude and behavior [15,17,18]. Thus, consumers who are more competent in purchasing foods would perceive more safety of foods. Previous studies have indicated that consumer competence is positively related to perceptions of food safety. Lee et al. [19] analyzed the effect of subjective consumer competence on consumer anxiety across food sectors and found that the higher subjective consumer competence was, the lower consumer anxiety about food hazards for imported fresh foods. They also found that subjective consumer competency negatively influenced consumer anxiety about food hazards for processed foods. In other words, as subjective consumer competence increased, consumer anxiety about hazards of processed foods decreased. Unexpectedly, they also found that subjective consumer competence had no significant impact on consumer anxiety about hazards of domestic fresh foods. On the other hand, Suh and Lim [20] investigated the effect of subjective consumer competence on consumer anxiety about food consumption and found that subjective consumer competence negatively influenced consumer anxiety about domestic and imported agricultural produce, seafood, livestock products, and fresh foods. In other words, as subjective consumer competence increased, consumer anxiety about these foods decreased. However, subjective consumer competence had no effect on consumer anxiety about frozen, instant, and processed foods. Thus, based on the knowledge-deficit model and previous findings, we expect the following:

**H1:**  *Competence in using food labels is positively related to perceived safety of (a) imported and (b) domestic foods*.

**H2:**  *Competence in using food information is positively related to perceived safety of (a) imported and (b) domestic foods.*

### 3.2. The Relationship between Perceptions of Food Safety and Satisfaction with Food-Related Consumer Policies

Food-related consumer policies are designed to protect consumers and improve the quality of food-related life [27]. Although few studies have explored any association of food safety with consumer policies in the food sector, it seems that food concerns lead consumers to support food policy [30,31]. Lee et al. [23] found that perceived food safety and trust in food labeling positively affected satisfaction with food-related consumer policies. On the other hand, unfavorable experiences toward foods and safety concerns about home meal replacements negatively affected satisfaction with food-related consumer policies [23]. Based on the above findings, we expect the following:

**H3:**  *Perceived safety of (a) imported and (b) domestic foods is positively related to satisfaction with food-related consumer policies*.

### 3.3. The Relationship between Perceptions of Food Safety and Satisfaction with Food-Related Life

A number of previous studies have examined the relationship between consumer anxiety about food hazards and satisfaction with food-related life. Lee et al. [19] found that consumer anxiety about hazards of domestic and imported fresh foods negatively affected satisfaction with food-related life. On the other hand, Suh and Lim [20] examined differences by household type in how consumer anxiety about food consumption affected satisfaction with food-related consumption. They found no relationship between food consumption anxiety and satisfaction with food-related consumption for single-person households. However, for multi-person households, satisfaction with food-related consumption was negatively affected by food consumption anxiety for domestic foods (agricultural produce, seafood, and livestock products). Liu and Grunert [32] identified three consumer segments based on Chinese elderly people’s beliefs about food health, safety, freshness, and taste. The segment highest in worry about food safety was also the lowest in satisfaction with food-related life. Based on the results from previous studies, we expect the following:

**H4:**  *Perceived safety of (a) imported and (b) domestic foods is positively related to satisfaction with food-related life*.

### 3.4. The Relationship between Satisfaction with Food-Related Consumer Policies and Satisfaction with Food-Related Life

Few studies have examined the relationship between satisfaction with food-related consumer policies and satisfaction with food-related life. However, some studies have confirmed that satisfaction with public policies significantly affects life satisfaction in other areas. Lee [33] classified the factors that influence life satisfaction for disabled people living in poverty into demographic, economic, social, physical, and policy-related factors and found that benefits from policy-related factors (e.g., satisfaction with welfare policies) were significant determinants of their life satisfaction. Ko and Lee [34] found that a job creation project had a significant policy effect on life satisfaction for the elderly: the higher the policy effect of the job creation project, the greater the life satisfaction for the elderly. Additionally, Whiteley et al. [35] analyzed monthly national surveys conducted in Britain and reported that public policy outcomes (e.g., health-care, financial security, and employment) significantly affected life satisfaction. On the basis of these findings, we expect the following:

**H5:**  *Satisfaction with food-related consumer policies is positively related to satisfaction with food-related life*.

## 4. Research Methods

### 4.1. Data Collection

We analyzed data from the “2019 Consumer Behavior Survey for Food,” which has been conducted every year since 2013 by the Korea Rural Economic Institute (KREI). The KREI conducts these multi-faceted surveys annually to enhance consumer satisfaction and competitiveness among food suppliers through identifying consumers’ food purchasing behaviors, changes in food preferences, and satisfaction with food policies of the government. By applying the stratified sampling method, the institute recruits primary household food shoppers with ages ranging from 18 to 75 and other adult and youth household members aged 13 to 75. The survey is usually administered between May and August every year. The primary food shopper in a household is interviewed face-to-face, while other household members participate in the survey by choosing either self-administered paper-and-pencil surveys or online surveys. For the 2019 survey, a total of 3968 households were contacted and 3337 households participated in the survey. For the current study, we analyzed the responses of the primary food shoppers of their households and other adult household members aged 18 to 75.

### 4.2. Measures

In this study, we examined the relationships between the constructs, including consumer competence in purchasing foods (i.e., competence in using food labels and competence in using food information), perceptions of food safety (i.e., perceived safety of imported foods and domestic foods), satisfaction with food-related consumer policies, and satisfaction with food-related life. All the scale items for the constructs were adopted from the questionnaire used in the “2019 Consumer Behavior Survey for Food.” Consumer competence in purchasing foods was subdivided into competence in using food labels (CUFL) and competence in using food information (CUFI) in this study. CUFL was assessed with an eight-item measure and CUFI with a six-item measure. Respondents were asked to indicate their level of agreement or disagreement with each item listed on a 5-point Likert-type scale, ranging from “strongly disagree” (1) to “strongly agree” (5).

The eight items for CUFL were as follows: “The quality of foods varies depending on the country of origin,” “I check the country of origin when purchasing foods,” “When purchasing foods, the GAP (Good Agricultural Practices) and HACCP (Hazard Analysis and Critical Control Point) certification can help me choose safe ones,” “In order to select safe foods, I purchase GAP and HACCP certified products first,” “The label information of foods on the packages and store shelves, such as location, content, and grade of the products, is all important information for product selection,” “I always carefully check the items marked on the packages and store shelves when purchasing foods,” “Nutrient ingredients are important when selecting foods,” and “I decide to purchase foods by referring to the nutritional information table.” The six items for CUFI applied were: “I am well aware of how to search for the information I need purchasing foods,” “I purchase foods after carefully comparing the relevant information such as the place of purchase, price, and quality,” “It is necessary to distinguish objective and accurate information among various information related to foods,” “For healthy consumption of foods, I use objective and accurate information to select desirable food,” “Information related to foods provided by the government and public organizations is very helpful in improving food-related life,” and “In everyday life, I use a lot of information related to the purchase of foods and food-related life.”

Perceived safety of imported foods (PSIF) and domestic foods (PSDF) were measured for 12 imported and 7 domestic foods. Respondents were asked, “Please indicate how safe you consider the following types of imported foods: grain, vegetables, fruit, beef (U.S. and Australian), European pork, Brazilian chicken, seafood (Japanese, Chinese, and European), processed food, and nuts.” Furthermore, respondents were asked, “Please indicate how safe you consider the following types of domestic foods: grain, vegetables, fruit, meat, seafood, processed foods, and nuts.” The responses were measured on a 5-point Likert-type scale, ranging from “very unsafe” (1) to “very safe” (5).

Food-related consumer policies are composed of five domain-specific areas, including policies relating to food safety, food-related education and public relations, food price/trade, food labeling, and compensation for consumers affected by unsafe food. To assess satisfaction with food-related consumer policies (SWFCP), respondents were asked “To what extent are you satisfied with the government’s food-related consumer policies?” Their satisfaction level with each policy category was measured on a scale of 0 to 100. Satisfaction with food-related life (SWFL) was measured using one question: “How satisfied are you with your food-related life?” The responses were measured on a 5-point Likert-type scale, ranging from “very dissatisfied” (1) to “very satisfied” (5).

## 5. Analyses and Results

### 5.1. Sample Characteristics

The sample included 6176 respondents who were primary food shoppers for their household and other adult household members. However, if respondents answered “don’t know” or “no opinion” for the items measuring perceived safety of imported and domestic foods, their responses were excluded from the analysis, leaving a final sample of 5869 respondents. Of the 5869 respondents, 44.9% were male and 55.1% were female. The ages in the sample ranged from 19 to 74, with an average age of 48.5 years. The largest age group (26.5%) was 50–59 years old, followed by 40–49 years old (23.1%). For education level, high school graduates (including college students) were the largest group (50.9%), followed by two-year or four-year college graduates with 35.1%. For average gross monthly household income, the largest group (19.0%) earned less than KRW 4 to 5 million (USD 3322.12 to 4152.65; EUR 2915.45 to 3644.31), followed by 18.2% earning less than KRW 5 to 6 million (USD 4152.65 to 4983.18; EUR 3644.31 to 4373.17). More details of the demographic characteristics of the sample are shown in Table 1.

### 5.2. Results of the Measurement Model

Because the constructs CUFL, CUFI, PSIF, and PSDF were represented by a large number of items (six to 12 items), it was difficult to analyze these constructs using structural equation modeling (SEM). Thus, we constructed a measurement model with partial disaggregation. In this method, the items relating to each of these constructs were randomly aggregated so that two or three composite indicators were created for each construct. Partial disaggregation is often used to reduce higher levels of measurement errors in complex SEM [36,37]. In this study, the measurement model included composite indicators with four latent constructs (i.e., CUFL, CUFI, PSIF, and PSDF) and individual indicators with two latent constructs (i.e., SWFCP and SWFL).

The validity of the measurement model was assessed through confirmatory factor analysis (CFA). The parameters of the model were estimated by maximum likelihood method using the AMOS program. The fit values of the measurement model were within acceptable levels for all values except the chi-square test (Table 2). Although the chi-square test was significant (*p* < 0.001), the fit values for the goodness-of-fit index (GFI), the comparative fit index (CFI), and the non-normed fit index (NNFI) were all greater than 0.90, and the root mean square error of approximation (RMSEA) value was smaller than 0.06, indicating a good model fit [38]. Because the chi-square test is sensitive to sample size, other fit values such as GFI, CFI, NNFI, and RMSEA more properly reflect model fit in a study with a large sample size [39].

Next, convergent validity of the measures was investigated. All the factor loadings were above 0.70, which were statistically significant (*p* < 0.001). Cronbach’s alpha (α) and composite reliability (CR) were also greater than a threshold value of 0.70 (α = 0.789 to 0.948; CR = 0.818 to 0.949). The average variance extract (AVE) exceeded 0.50 for each construct (AVE = 0.601 to 0.788), indicating that each construct accounted for more than 50% of the variance in the underlying construct. Thus, convergent validity was established (Table 3).

For discriminant validity, we used the criterion suggested by Fornell and Larcker [40]. The square root of the AVE of each construct exceeded its correlations with all other constructs. Accordingly, discriminant validity was also established (Table 4).

### 5.3. Results of Hypothesis Testing

The structural model with a series of causal relationships between the constructs was constructed and tested. The effects of control variables (i.e., age and education) were also analyzed. Although the chi-square test was significant (*p* < 0.001), other goodness-of-fit values were good (Table 5).

The results of hypothesis testing are summarized in Table 6. Interestingly, contrary to our expectations, CUFL had a significant but negative effect on PSIF (*γ* = −0.088, *p* < 0.001). As consumers’ competence in using food labels increases, imported foods are perceived as less safe. Thus, H1a was not supported. However, CUFL had a significant positive effect on PSDF (*γ* = 0.136, *p* < 0.001). These findings confirmed the proposed relationship, supporting H1b. As consumers’ competence in using food labels increases, their perceived safety of domestic foods become more positive. H2 was fully supported. CUFI had a significant, positive effect on PSIF (*γ* = 0.271, *p* < 0.001) and PSDF (*γ* = 0.119, *p* < 0.001). As consumers’ competence in using food information improves, both imported foods and domestic foods are perceived as safer.

As expected, the relationship between PSIF and SWFCP was significant (*β* = 0.213, *p* < 0.001). However, the effect of PSDF on SWFCP was not significant (*β* = 0.004, *p* = 0.769). Thus, H3a was supported, but H3b was not. The more positively consumers’ perceived safety of imported foods, the higher their satisfaction was with food-related consumer policies. H4 was partially supported. While the relationship between PSIF and SWFL was not significant (*β* = 0.022, *p* = 0.121), PSDF had a significant positive effect on SWFL (*β* = 0.140, *p* < 0.001). H4a was not supported, while H4b was supported. The more positively consumers’ perceived safety of domestic foods, the higher their satisfaction was with food-related life. Lastly, H5 was supported because there was a significant positive relationship between SWFCP and SWFL (*β* = 0.053, *p* < 0.001). As consumers are more satisfied with food-related consumer policies, they are also more satisfied with food-related life.

The control variables of age and education showed significant results. Age was positively related to SWFL (*γ* = 0.072, *p* < 0.001): as age increases, satisfaction with food-related life increases. On the other hand, education was negatively related to PSDF (*γ* = −0.050, *p* < 0.001): as education level increases, perceived safety of domestic foods decreases. Meanwhile, age had no significant effects on PSIF (*γ* = −0.011, *p* = 0.407) and SWFCP (*γ* = 0.016, *p* = 0.227) and education had no significant effects on SWFCP (*γ* = −0.009, *p* = 0.506) and SWFL (*γ* = −0.013, *p* = 0.315).

## 6. Discussion and Implications

According to the knowledge-deficit model, a person’s knowledge is an important factor that induces change in a person’s attitude and behavior, and a certain level of a person’s competence leads to reasonable attitudes or behaviors [14,15,16]. Based on the knowledge-deficit model, the current study examines how consumer competence in purchasing foods affects perceptions of food safety, satisfaction with food-related consumer policies and satisfaction with food-related life. In the following section, we discuss the findings of this study and how they can be applied to the development of food-related consumer policies.

First, all but one of the proposed relationships between consumer competence in purchasing foods and perceptions of food safety were supported. The exception was competence in using food labels, which is negatively related to perceived safety of imported foods. This outcome is inconsistent with the predictions of the knowledge-deficit model and the previous findings of Suh and Lim [20] and Lee et al. [19], who found that people with higher levels of consumer competence perceived lower levels of anxiety about imported foods. This discrepancy may exist because consumers who are highly competent in using labels in purchasing foods may examine food labels of imported foods very carefully and may doubt the safety of imported foods if information shown on the label is dissimilar to that of the food labels of domestic foods or if necessary information is not shown. For this reason, the government should be actively involved in establishing and implementing food labeling policies of imported foods to ensure consumers’ perceptions of food safety. For example, the government should monitor whether country of origin and sufficient amounts of information are supplied on the food labels of imported foods. Nevertheless, most of the results supported the proposed relationship. Therefore, our study offers implications for increasing consumers’ perception of food safety. The implementation of well-designed consumer education and public relations programs will strengthen consumer competence in purchasing foods, ultimately resulting in improved perceptions of food safety. For example, consumer education programs need to repeatedly deliver simple and easy messages rather than complex information, and these programs should be available via various types of media (video and visual materials) that consumers can easily access [41].

Second, the relationship between perceptions of food safety and satisfaction with food-related consumer policies depends on whether foods are imported or domestic. Safety perception of imported foods significantly affects satisfaction with food-related consumer policies, but safety perception of domestic foods does not. This finding indicates that satisfaction with food-related consumer policies is based solely on the perceived safety of imported foods. Thus, when establishing food-related consumer policies, government ministries should develop detailed plans to improve perceived safety of imported foods. For example, to promote satisfaction with food-related consumer policies, the government could implement a policy to strengthen consumer education and public relations activities on the topic of recently introduced imported foods and food hazards of imported foods that may still be unfamiliar to consumers.

Third, food origin also affected the relationship between perceptions of food safety and satisfaction with food-related life. Satisfaction with food-related life is significantly influenced by perceived safety of domestic foods but not by perceived safety of imported foods. This outcome is similar to the findings of Suh and Lim [20], who found an inverse relationship between consumption anxiety for domestic foods and satisfaction with food-related life. However, consumption anxiety for imported foods had no relationship with satisfaction with food-related life. These results show how important perceived safety of domestic foods is to Korean consumers’ satisfaction with food-related life. Thus, the government should strive to secure Korean consumers’ perception that domestic foods are safe, thus enhancing their satisfaction with food-related life.

Fourth, satisfaction with food-related consumer policies is significantly connected with satisfaction with food-related life. The government’s food-related consumer policies are being applied to many aspects of food-related life, ultimately resulting in higher satisfaction with food-related life. Thus, the government should establish and implement food-related consumer policies, which can improve satisfaction with food-related life.

This study has some limitations that should be taken into consideration for future study. The study revealed that competence in using food labels was inversely associated with perceived safety of imported foods, a finding which is inconsistent with the knowledge-deficit model. Thus, further studies are needed to explore the relationship between other dimensions of consumer competence (e.g., eating habit competence and food damage relief competence) and safety perceptions of foods.

It should also be noted that some constructs had many measurement items, possibly causing a high level of random errors in structural equations. Thus, we used the partial disaggregation method when constructing the measurement model in SEM. However, this method could not provide as detailed an analysis as the total disaggregation method, which is a traditional SEM approach [36]. Thus, future research could modify or refine the scale items and reduce the number of the items per latent construct to three to five, the ideal number of items per latent construct [42]. Furthermore, satisfaction with food-related life was evaluated with one single item. Thus, subsequent studies could adopt a scale composed of multiple items for satisfaction with food-related life, such as the scale developed by Grunert et al. [28].

A sample drawn from only one country (i.e., South Korea) was utilized to test the model in this study. Thus, the study results may not be applicable to consumers in other countries, such as Western countries. Consumers in Western countries are dissimilar to those in Eastern countries in many aspects, such as culture, consumer competence, or perceptions of food safety. Thus, future research could replicate the current study with samples in other countries and provide a further cross-validation of the current study’s results.

## Figures and Tables

**Table 1:** Sample characteristics.

Demographic		Frequency	%
**Gender**	Male	2636	44.9
	Female	3233	55.1
Age	19–29	731	12.5
	30–39	795	13.5
	40–49	1353	23.1
	50–59	1556	26.5
	60–69	1110	18.9
	70–74	324	5.5
Education	Middle school or less	804	13.7
	High school (including college students)	2988	50.9
	Bachelor’s degree (including associate degree and graduate students)	2061	35.1
	Master’s degree or higher	16	0.3
Monthly household income (KRW ^1^)	Under KRW 1 million	294	5.0
	KRW 1 million–KRW 2 million	641	10.9
	KRW 2 million–KRW 3 million	882	15.0
	KRW 3 million–KRW 4 million	1050	17.9
	KRW 4 million–KRW 5 million	1114	19.0
	KRW 5 million–KRW 6 million	1071	18.2
	KRW 6 million or more	817	14.0
Total		5869	100

Note: ^1^ KRW = Korean Won. KRW 1,000,000 (USD 829.96; EUR 830.53).

**Table 2:** Fit indices of measurement model.

χ^2^	GFI	CFI	NNFI	RMSEA
1698.215	0.971	0.979	0.973	0.044

Note: GFI = goodness-of-fit index; CFI = comparative fit index; NNFI = non-normed fit index; RMSEA = root mean square error of approximation.

**Table 3:** Analysis of the measurement model.

Construct	Item	Standardized Loading	*t*-Value	CR	AVE
Competence in using food labels	CUFL_1	0.931	92.776 ***	0.911	0.777
	CUFL_2	0.969	99.496 ***		
	CUFL_3	0.724	63.580 ***		
Competence in using food information	CUFI_1	0.821	71.982 ***	0.839	0.634
	CUFI_2	0.783	67.339 ***		
	CUFI_3	0.784	67.419 ***		
Perceived safety of imported foods	PSIF_1	0.821	72.991 ***	0.877	0.642
	PSIF_2	0.882	80.640 ***		
	PSIF_3	0.757	64.380 ***		
	PSIF_4	0.736	61.868 ***		
Perceived safety of domestic foods	PSDF_1	0.777	62.998 ***	0.818	0.601
	PSDF_2	0.799	65.056 ***		
	PSDF_3	0.748	60.405 ***		
Satisfaction with food-related consumer policies	SWFCP_1	0.899	88.124 ***	0.949	0.788
	SWFCP_2	0.893	86.649 ***		
	SWFCP_3	0.890	86.591 ***		
	SWFCP_4	0.869	82.742 ***		
	SWFCP_5	0.888	86.229 ***		
Satisfaction with food-related life	SWFL_1	—	108.333 ***	—	—

Note: CR = composite reliability; AVE = average variance extracted. *** *p* < 0.001.

**Table 4:** Discriminant validity.

Construct	Mean	S.D.	(1)	(2)	(3)	(4)	(5)	(6)
(1) CUFL	3.612	0.544	0.881					
(2) CUFI	3.440	0.647	0.743	0.796				
(3) PSIF	3.397	0.707	0.111	0.202	0.801			
(4) PSDF	4.029	0.511	0.218	0.209	0.083	0.775		
(5) SWFCP	73.189	14.435	0.088	0.146	0.210	0.017	0.888	
(6) SWFL	3.631	0.547	0.143	0.137	0.038	0.137	0.061	1

Note: Diagonal elements are AVE by each construct. Lower diagonal elements are the squared correlation coefficient. CUFL = competence in using food labels; CUFI = competence in using food information; PSIF = perceived safety of imported foods; PSDF = perceived safety of domestic foods; SWFCP = satisfaction with food-related consumer policies; SWFL = satisfaction with food-related life. CUFL, CUFI, PSIF, PSDF, and SWFL are rated on a 5-point Likert-type scale; SWFCP is rated on a score of 0–100.

**Table 5:** Fit indices of structural model.

χ^2^	GFI	CFI	NNFI	RMSEA
3517.426	0.947	0.955	0.946	0.057

Note: GFI = goodness-of-fit index; CFI = comparative fit index; NNFI = non-normed fit index; RMSEA = root mean square error of approximation.

**Table 6:** Results of hypotheses tests.

Hypothesized Relationship	Standardized Estimates	*t*-Value	Hypothesis Supported
H1a: CUFL → PSIF	−0.088	−3.740 ***	No
H1b: CUFL → PSDF	0.136	5.606 ***	Yes
H2a: CUFI → PSIF	0.271	10.793 ***	Yes
H2b: CUFI → PSDF	0.119	4.642 ***	Yes
H3a: PSIF → SWFCP	0.213	15.042 ***	Yes
H3b: PSDF → SWFCP	0.004	0.294	No
H4a: PSIF → SWFL	0.022	1.551	No
H4b: PSDF → SWFL	0.140	9.731 ***	Yes
H5: SWFCP → SWFL	0.053	3.893 ***	Yes

Note: CUFL = competence in using food labels; CUFI = competence in using food information; PSIF = perceived safety of imported foods; PSDF = perceived safety of domestic foods; SWFCP = satisfaction with food-related consumer policies; SWFL = satisfaction with food-related life. *** *p* < 0.001.
